# From gut to brain: short-term ketogenic diet alleviates status epilepticus-induced cognitive deficits in rats

**DOI:** 10.3389/fphys.2026.1752371

**Published:** 2026-04-28

**Authors:** Yimin Chen, Wanyin Xu, Qun Hou, Yan Jiang

**Affiliations:** 1 Department of Special Inspection, Affiliated Mental Health Center & Hangzhou Seventh People’s Hospital, Zhejiang University School of Medicine, Hangzhou, China; 2 Department of Nutrition, The First Affiliated Hospital of Zhejiang Chinese Medical University (Zhejiang Provincial Hospital of Chinese Medicine), Hangzhou, China; 3 Department of Neurology, The First Affiliated Hospital of Zhejiang Chinese Medical University (Zhejiang Provincial Hospital of Chinese Medicine), Hangzhou, China

**Keywords:** cognitive function, epilepsy, ketogenic diet, microbiota-gut-brain axis, short-chain fatty acids

## Abstract

**Backgrounds:**

Cognitive impairment is common in epilepsy. Ketogenic diets (KD) are shown to improve cognitive function in patients with drug-resistant epilepsy over the long term. It is believed that the microbiota-gut-brain axis affects brain function and behavior. However, the effects and mechanisms of short-term KD use on cognition remain unclear. By studying the microbiota-gut-brain axis, we aim to examine the effects of short-term KD use on cognition in an epilepsy rat model.

**Methods:**

Rats with epilepsy were fed a KD or a normal diet (ND) for 4 weeks. Following the lithium-pilocarpine-induced status epilepticus (SE), an assessment of cognitive function was performed using the Morris Water Maze (MWM) test. Fecal short-chain fatty acids (SCFAs), serum amino acids, and neurotransmitters were analyzed in accordance with gut microbiota composition.

**Results:**

On the probe trials of the MWM, rats in the KD group showed significantly shorter escape times (*P* < 0.001) and spent more time in the target quadrant (*P* < 0.05) compared to rats in the ND group. KD was associated with reduced microbial richness compared to ND, as well as distinct differences in gut microbiota composition across phyla, families, and genera. The KD group had significantly lower levels of fecal SCFAs (*P* < 0.05 for isobutyric acid and isovaleric acid; *P* < 0.01 for butyric acid; *P* < 0.0001 for acetic acid, propionic acid, valeric acid, and caproic acid). Serum amino acids and neurotransmitters also exhibited corresponding alterations. The KD group showed significantly elevated levels of norepinephrine, histamine, and threonine (all *P* < 0.05), dopamine, 5-hydroxytryptamine, acetylcholine, and serine (all *P* < 0.01), and glutamate (*P* < 0.001). Conversely, levels of arginine, phenylalanine, methionine, and asparagine (all *P* < 0.01), tryptophan, kynurenine, and ornithine (all *P* < 0.001), and lysine and tyrosine (both *P* < 0.0001) were significantly reduced.

**Conclusions:**

In epileptic rats, short-term use of the KD may modulate gut microbiota and enhance cognition. Shifts in gut microbiota are associated with changes in neurotransmitters and amino acids. Further investigation is warranted into the microbiota-gut-brain axis as a biomarker for cognitive improvement in epilepsy.

## 1 Introduction

Cognitive and behavioral problems are experienced by 20% to 50% of patients with epilepsy, including difficulties with memory, language, visual and spatial abilities, executive function, calculation, and comprehension (Catalán-Aguilar et al., 2025). Generally, these issues are associated with recurrent epileptic seizures, antiseizure medications, and interictal epileptiform activities (Auvin, 2022), which can be significantly disabling and potentially even life-threatening (Singh and Sander, 2020).

The ketogenic diet (KD), a high-fat, low-carb regimen, has been used to treat epilepsy since the 1920s (Lambrechts et al., 2017). However, cognitive outcomes under the KD are remarkably variable across different animal species, seizure models, and feeding durations (Hori et al., 1997; Su et al., 2000; Jiang et al., 2016). The cognitive benefits of KD in patients with epilepsy are also inconsistent (van Berkel et al., 2018). Even in studies reporting cognitive improvements, it remains unclear whether such benefits derive from seizure control per se or represent direct effects of the diet (Patel et al., 2010; DM et al., 2016). It has been shown, however, that KD improves cognitive function in conditions like Alzheimer’s and autism (Ozler and Sanlier, 2025; Szabo-Reed and Key, 2025), suggesting that it may have effects on cognition that extend beyond seizure reduction.

The microbiota-gut-brain axis has emerged as a candidate mechanism underlying cognitive benefits of KD (Zhang et al., 2018; Olson et al., 2021). This axis encompasses bidirectional communication between host metabolism and gut microbiota, including microbial metabolites such as short-chain fatty acids (SCFAs) and amino acid-derived neurotransmitter precursors (Dahlin et al., 2005; Olson et al., 2018; Ferraris et al., 2021). However, it remains unclear whether KD modulates brain function through direct effects on host metabolism or indirectly via gut microbiota and their metabolites. Therefore, a combined assessment of cognition, gut microbiota, and blood amino acids offers a preliminary approach to analyzing whether KD exerts these effects through direct or microbiota-mediated peripheral metabolic pathways.

Clinically, KD is generally studied over a long period, typically 12 weeks or more. However, some clinical situations, such as status epilepticus and acute cognitive impairment, require rapid KD interventions. KD can rapidly induce ketosis and regulate physiological metabolism, providing immediate metabolic support and neuroprotection (McDonald and Cervenka, 2020). Moreover, short-term KD findings can guide the introduction of modified support strategies in clinical settings, facilitating patients’ transitions to long-term KD. However, the mechanisms and clinical value of short-term KD interventions, typically within 4 weeks, have not yet been fully explored.

This study aims to explore the short-term effects of KD on cognitive function in SE-associated acute cognitive impairment, examine associated changes in gut microbiota and blood amino acids, and investigate whether microbial or host metabolic factors contribute to these effects. We hypothesize that the microbiota-gut-brain axis may contribute to KD’s cognitive effects through microbial metabolites such as SCFAs and neurotransmitter precursors.

## 2 Materials and methods

### 2.1 Animals and diet

Animal research was approved by the Laboratory Animal Management and Ethics Committee at Zhejiang Chinese Medical University (IACUC-20221017-10). All studies were carried out in accordance with local and international guidelines for animal care and conform to ARRIVE guidelines and regulations. Sample size for behavioral testing was estimated by *a priori* power analysis (G*Power 3.1, α = 0.05, power = 0.8, two-tailed, d = 1.2). In accordance with the 3Rs principle and based on our pilot data showing differential mortality rates (ND: 30%–40%; KD: 10%–20%), we allocated n = 15 to the ND group and n = 10 to the KD group.

Postnatal day 21 (P21) male Sprague Dawley rats (40–60 g) from Shanghai Slake Experimental Animal Co., Ltd. were acclimatized for 1 week in an SPF facility. At P28, the rats were fed a ketogenic diet (KD; ketogenic ratio 3:1, Shenzhen Zeneca Biotechnology Co., Ltd.) or a normal diet (ND) for 4 weeks before model establishment. Blood glucose and beta-hydroxybutyrate (BHB) levels were measured weekly for 5 weeks using blood glucose and ketone test strips, encompassing both the modeling and behavioral testing periods (**Supplementary figure 1**).

### 2.2 The lithium-pilocarpine model

To induce seizures in both groups, lithium chloride (LiCl; 127 mg/kg, intraperitoneal) was administered. After a 24 h interval, scopolamine methyl bromide (1 mg/kg, intraperitoneal) was given. Thirty minutes later, pilocarpine (initial dose 30mg/kg, intraperitoneal) was injected (Smolensky et al., 2019). Seizure identification and quantification were performed using a modified Racine scale (stages 1–5), with stages 1–3 defined as focal seizures and stages 4–5 as generalized seizures (Jiang et al., 2012). If no sustained seizure of Racine grade IV or higher occurred within 30 minutes, 10 mg/kg pilocarpine was administered every 15 minutes until one occurred. To terminate status epilepticus, 5 mg/kg diazepam was injected one hour after seizure onset. An overview of the seizure burden during the lithium-pilocarpine model was recorded (**Supplementary figure 2**). Due to seizure-related mortality and exclusion during MWM pre-training, 7 rats per group completed behavioral testing. For gut microbiota analysis, 3 ND samples were excluded due to poor quality or insufficient material, resulting in n = 7 for KD and n = 4 for ND.

### 2.3 Behavioral tests

We conducted a MWM test to assess spatial memory and learning four days after pilocarpine injection, a time point selected to target the latent phase of the lithium-pilocarpine model when spontaneous seizures are minimal. The detailed MWM procedures have been provided in the **Supplementary Materials**.

Based on prior evidence that lithium-pilocarpine rats exhibit significant spatial memory deficits during the latent period compared to sham controls (Rice et al., 1998; Hort et al., 1999), the present study focused specifically on comparing cognitive outcomes between KD-treated and ND-treated epileptic rats to assess the dietary intervention effects.

### 2.4 Sample collection, detection and analysis

Rats were deeply anesthetized with 3% sodium pentobarbital (50 mg/kg body weight, Merck) via intraperitoneal injection for at least 10 min, followed by cervical dislocations to ensure humane euthanasia. For cardiac blood collection, the rats were positioned supine, their chest fur was shaved and disinfected, and blood was obtained using a 2 mL heparinized syringe and collected into lithium heparin tubes to prevent coagulation. Subsequently, the samples were then centrifuged at 1500 × g for 10 min at 4 °C to obtain plasma, which was stored at -80 °C until analysis. For fecal collection, the contents of the rectum were excised and placed in EP tubes. All samples were labeled, stored in a dry ice box, and transferred to a -80 °C freezer. Serum neurotransmitters, amino acids, fecal SCFAs, and 16S rRNA sequencing were analyzed by Suzhou PANOMIX Biomedical Tech Co., Ltd. (CMA certification number: 201910040237). Its methodologies have been previously published and validated, ensuring accuracy and reliability (Liu et al., 2023; Ji et al., 2025; Zhang F. et al., 2025).

### 2.5 Statistical analysis

The MWM test, serum neurotransmitters, amino acids, and fecal SCFAs were analyzed with SPSS Statistics 22.0 (IBM, Armonk, NY, USA). A two-way repeated measures ANOVA was conducted to examine the effects of diet and time on escape latency during hidden platform training, with pairwise comparisons performed using Bonferroni correction. For other MWM comparisons between two groups, an independent samples t-test or Mann-Whitney U test was used. 16S rRNA gene sequencing analysis, as well as alpha (α)- and beta (β)-diversity analyses, were performed using QIIME 2 (version 2019.4). Diversity indices (such as Shannon and Chao1) were used to evaluate the α-diversity of the samples. The significance of differences in α-diversity among groups was assessed using the Kruskal-Wallis rank sum test, followed by Dunn’s test for *post hoc* comparisons. β-diversity was assessed using non-metric multidimensional scaling (NMDS) and visualized using principal coordinate analysis (PCoA). β-diversity analyses were conducted using analysis of similarities (ANOSIM) with a permutation test to evaluate significant differences between sample groups. The ANOSIM statistic R ranges from -1 to 1, with R<0 indicating greater within-group than between-group differences, and R>0 indicating greater between-group than within-group differences. Linear discriminant analysis (LDA) effect size (LEfSe) was used to identify statistically differences in the relative abundance of taxa. Only LDA values >3 were considered significantly enriched. A p-value of less than 0.05 was considered statistically significant.

## 3 Results

### 3.1 Ketonemia and Seizure burden during the acute SE stage

Blood glucose and serum BHB levels were measured in the ND and KD groups. After week 1, KD induced persistent hypoglycemia and ketonemia. Blood glucose levels were lower in KD than in ND (*P* < 0.001), while serum BHB levels were higher in KD than in ND rats (*P* < 0.001) (**Supplementary Figures 1A, B**).

During the acute SE stage in the lithium-pilocarpine model, the latency to seizure, duration of seizure, and duration of generalized seizures were recorded. No significant differences were observed between the KD and ND group (all *P* > 0.05). (**Supplementary Figure S2**).

### 3.2 Water maze test

Swimming speed did not differ significantly between the two groups during visible platform training (**Supplementary Figure 3A**). During the 4-day hidden platform training period, rats in the KD group exhibited longer escape latencies compared to those in the ND group (*P* < 0.01). The escape latency for the ND group decreased with training, becoming significantly shorter on Day 3 (*P* < 0.01) than on Day 1. A decrease in latency was also observed in the KD group on Day 4 (*P* < 0.05) (**Supplementary Figure 3B**).

**Figure 1:**
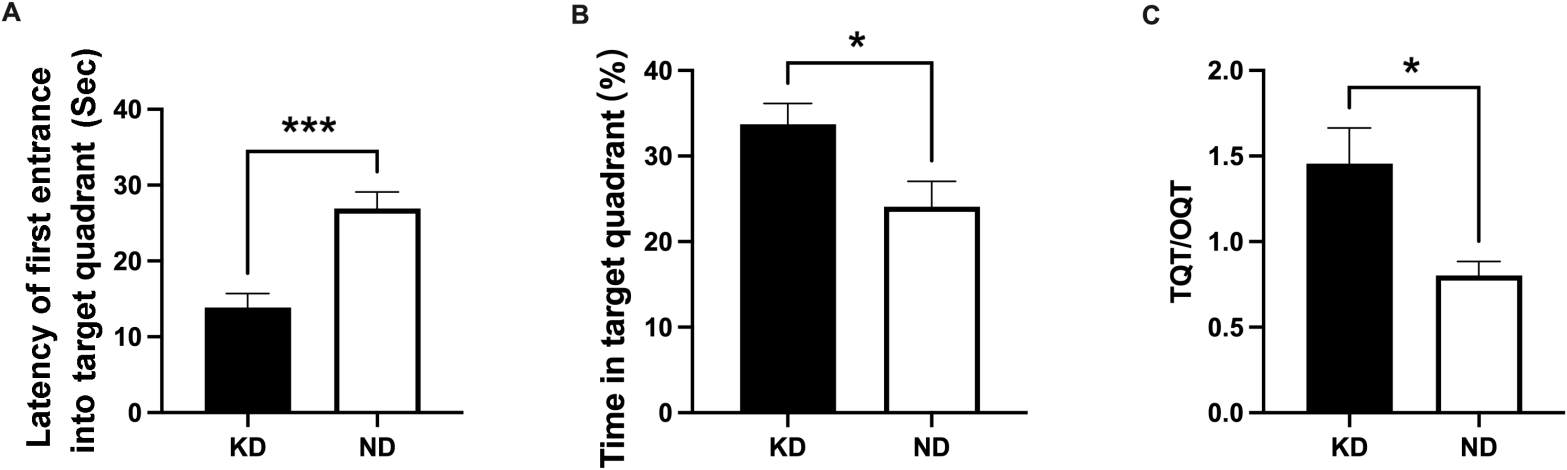
Morris Water Maze (MWM) test performance in the spatial probe trial. **(A)** Escape latency; **(B)** Retention time in the target quadrant; **(C)** TQT/OQT ratio = target quadrant time/opposite quadrant time. n=7 for the KD and ND group. *P<0.05, ***P<0.001 for comparisons between groups.

The spatial probe test results indicated no significant differences between the KD and ND groups in terms of swimming speed and platform crossings (all *P* > 0.05; **Supplementary Figure 3C**). However, the KD group exhibited significantly shortened escape latency (*P* < 0.001, **Figure 1A**) and spent more time in the target quadrant (*P* < 0.05, **Figures 1B, C**).

### 3.3 16S rRNA gene sequencing analysis

#### 3.3.1 Species composition

**Figure 2:**
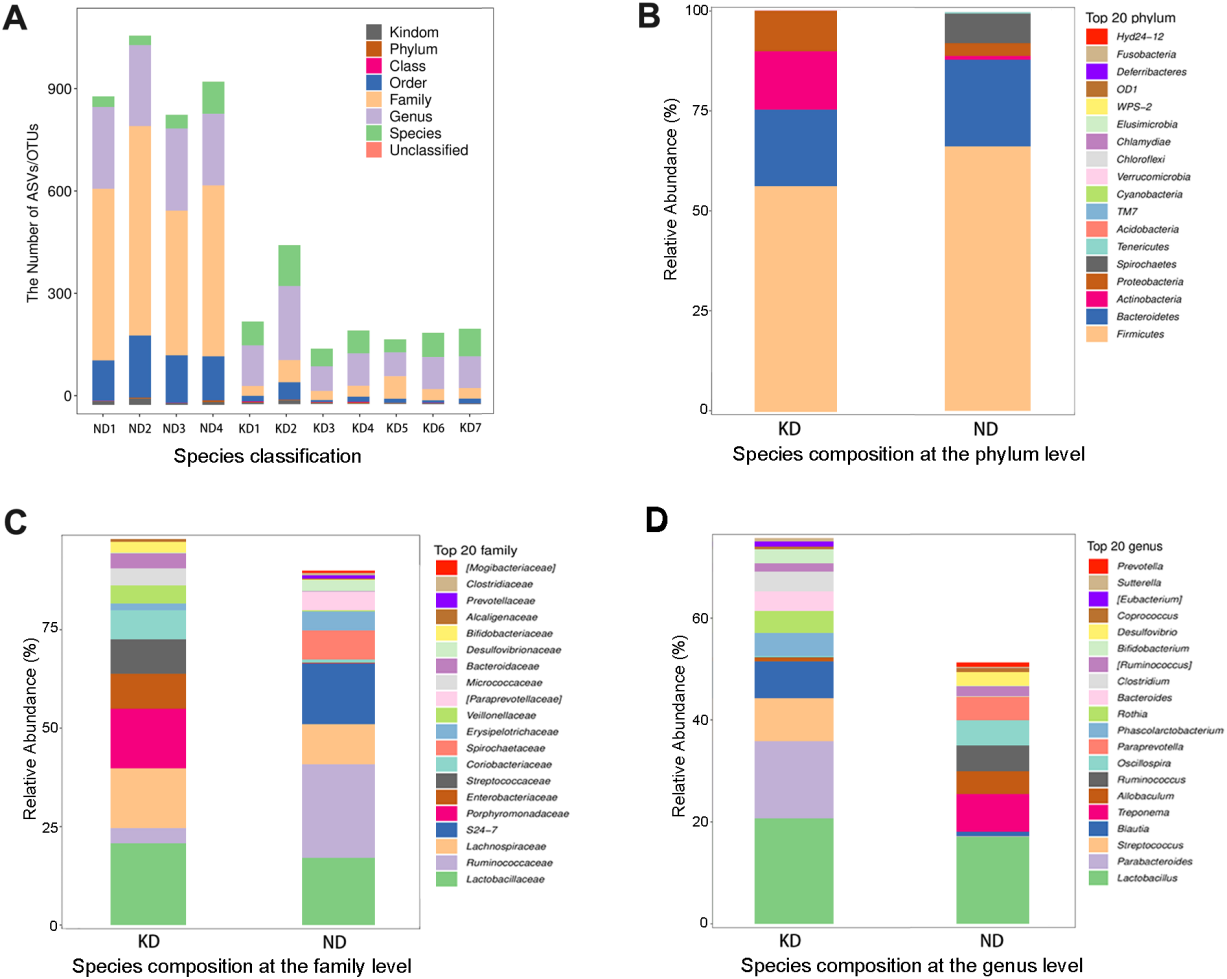
Gut microbiota species composition analysis. **(A)** Stacked bar chart of species classification in KD and ND Groups; **(B)** Phylum-Level Species comparison; **(C)** Family-Level Species comparison; **(D)** Genus-Level Species Comparison. n=7 for KD group, n=4 for ND group.

As illustrated in **Figure 2A**, the KD group exhibited a significantly lower total number of intestinal flora compared to the ND group.

At the phylum level, the types of bacterial species were similar between the two groups. However, the KD group showed a significant reduction in the abundance of *Firmicutes* and *Spirochaetes*, along with a slight decrease in *Bacteroidetes*. Conversely, the abundance of *Proteobacteria* and *Actinobacteria* was significantly higher in the KD group (**Figure 2B**).

Compared with the ND group at the family level, the KD group had a significant increase in the relative abundance of *Lactobacillaceae* and *Lachnospiraceae*, while *Ruminococcaceae* decreased significantly. The KD group also had higher levels of *Porphyromonadaceae*, *Streptococcaceae*, *Coriobacteriaceae*, and *Veillonellaceae*. In contrast, the ND group was dominated by *S24-7*, *Spirochaetaceae*, and *Erysipelotrichaceae* (**Figure 2C**).

**Figure 3:**
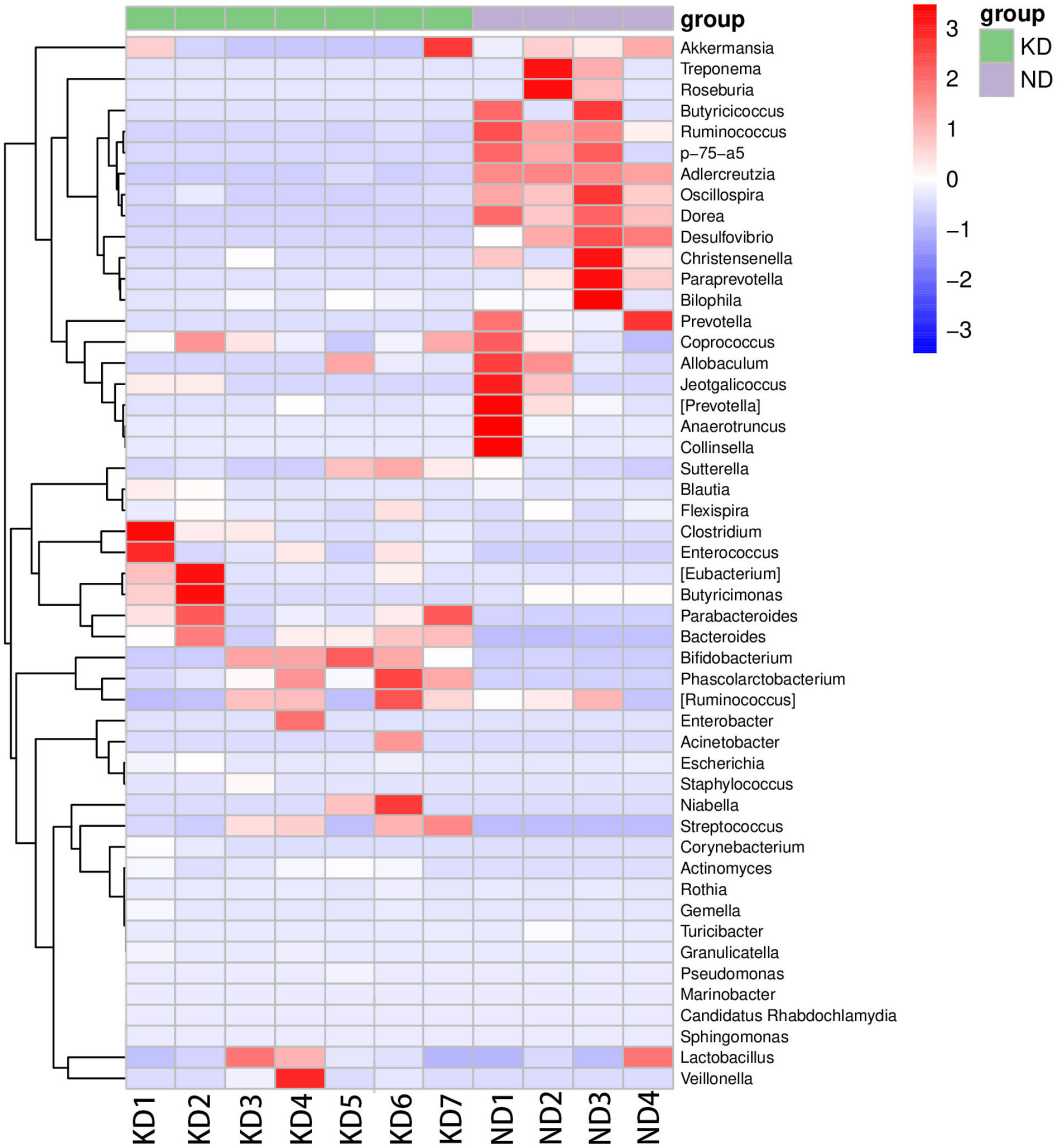
Heatmap of Genus-level Species Composition. The heatmap illustrates the differences in the abundance of various genera between the KD and ND groups. n=7 for KD group, n=4 for ND group. Red color blocks indicate a higher abundance of the genus in the sample compared to other samples, while blue color blocks indicate lower abundance.

At the genus level, *Lactobacillus* was common in both groups but was more abundant in the KD group. The KD group also had higher levels of *Parabacteroides, Streptococcus, and Blautia*. The ND group was characterized by *Treponema, Allobaculum, Ruminococcus, Oscillospira, Paraprevotella*, and *Desulfovibrio* (**Figure 2D**). A heatmap of species clustering showed clear differences in genus abundance between the KD and ND groups (**Figure 3**).

#### 3.3.2 α- and β-diversity analysis

**Figure 4:**
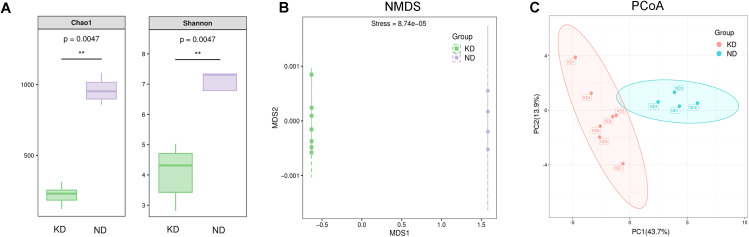
Diversity and abundance of gut microbiota. **(A)** Grouped boxplots of α-diversity indices: Chao1 indices reflect richness; Shannon indices reflect diversity. (**P < 0.01 for comparisons between groups); **(B)** β-diversity analysis assessed by Non-metric Multidimensional Scaling (NMDS); **(C)** β-diversity analysis visualized by Principal Coordinates Analysis (PCoA). n=7 for KD group, n=4 for ND group.

α- and β-diversity are crucial for evaluating species diversity. α-diversity reflects the diversity within a single ecosystem (Perzon and Ilan, 2025), and the main indicators, including Chao1 values and Shannon index, are significantly lower in the KD group compared to the ND group (both *P*<0.01, **Figure 4A**). β-diversity measures differences in microbial community composition between samples, based on changes in abundance or evolutionary relationships (Perzon and Ilan, 2025). Analysis showed significant differences in microbiota structure between the KD and ND groups, as shown in NMDS and PCoA plots (**Figures 4B, C**). ANOSIM confirmed that the differences between the two groups were statistically significant (R = 0.83, *P*<0.01).

#### 3.3.3 Species difference analysis

**Figure 5:**
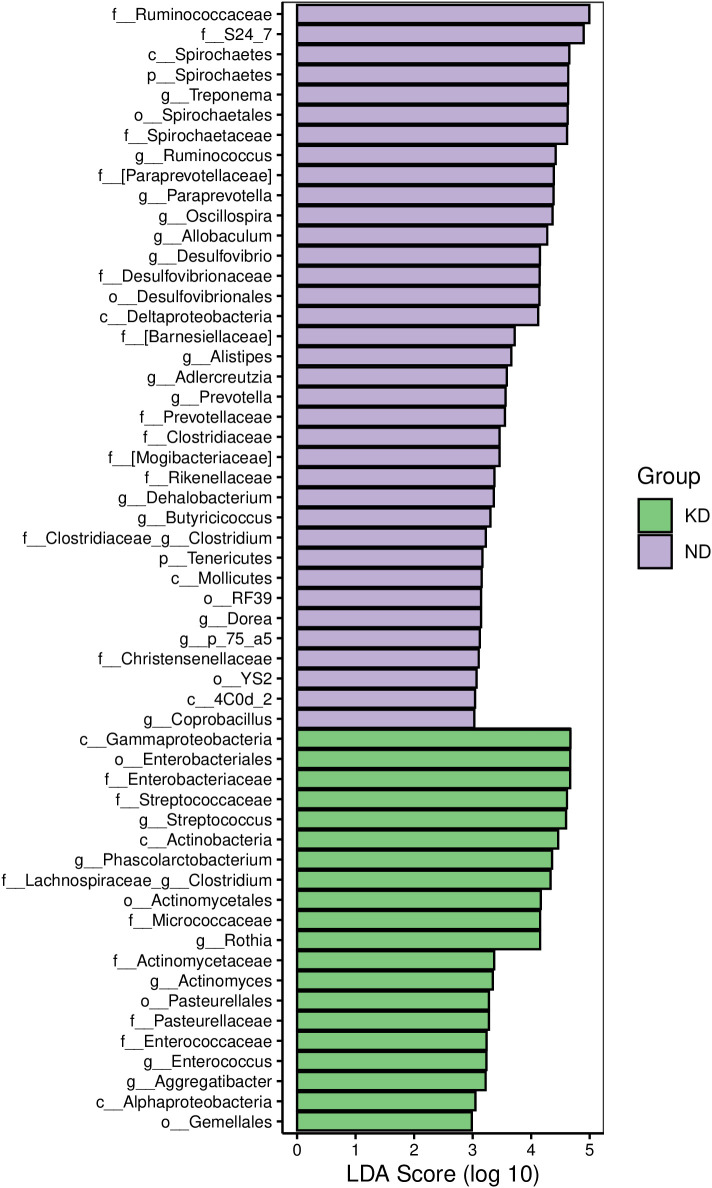
LDA effect size. The bar chart shows the LDA effect size (LEFSe) for microbial groups differing significantly between the KD and ND groups. Bar length indicates the LDA score logarithm, with green bars indicating enriched microbiota in the KD group and purple bars indicating enriched microbiota in the ND group. Only species with an LDA score > 3.0 are shown, indicating statistical significance (P < 0.05). n=7 for KD group, n=4 for ND group.

As shown in **Figure 5**, LEfSe analysis identified significant differences in microbial groups between the KD and ND groups. *Streptococcus, Phascolarctobacterium, Clostridium, Rothia, Actinomyces, Enterococcus, and Aggregatibacter* are identified as “marker species” in the gut microbiota of the KD group.

### 3.4 Fecal SCFAs

Compared with the ND group, fecal SCFAs in the KD group rats were significantly decreased for acetic acid (*P* < 0.0001), propionic acid (*P* < 0.0001), butyric acid (*P* < 0.01), isobutyric acid (*P* < 0.05), valeric acid (*P* < 0.0001), isovaleric acid (*P* < 0.05), and caproic acid (*P* < 0.0001) (**Table 1**).

**Table 1:** Fecal short-chain fatty acid (x̄ ± s, μg/mL).

SCFAs	KD (n=7)	ND (n=4)	*T value*	*P value*
Acetic acid	55.21 ± 28.51^****^	255.00 ± 25.00	*11.64*	*<0.0001*
Butyric acid	35.31 ± 14.51^**^	141.7 ± 76.39	*3.719*	*0.0048*
Propionic acid	13.76 ± 9.48^****^	73.51 ± 10.07	*9.845*	*<0.0001*
Valeric acid	0.30 ± 0.29^****^	7.82 ± 2.41	*8.493*	*<0.0001*
Isobutyric acid	0.19 ± 0.30^*^	3.76 ± 3.08	*3.170*	*0.0114*
Isovaleric acid	0.08 ± 0.14^*^	3.09 ± 3.19	*2.602*	*0.0287*
Caproic acid	0.01 ± 0.01^****^	3.484 ± 0.92	*10.42*	*<0.0001*

Compared with the ND group, *P<0.05, **P<0.01, ***P<0.001, ****P<0.0001.

### 3.5 Serum neurotransmitters and Amino Acids

Compared with the ND group, KD group showed significant increases in several serum neurotransmitters, including glutamic acid (Glu, *P* < 0.001), histamine (His, *P* < 0.05), HisA (*P* < 0.05), norepinephrine (NE, *P* < 0.05), 5-hydroxytryptamine (5-HT, *P* < 0.01), acetylcholine (Ach, *P* < 0.01), and dopamine (DOPA, *P* < 0.01). Also, the KD group had significant increases in serum amino acids such as serine (Ser, *P* < 0.01) and threonine (Thr, *P* < 0.05) (**Table 2**).

**Table 2:** Serum neurotransmitter and Amino Acids (x̄ ± s, μg/mL).

NTs/AAs	KD (n=7)	ND (n=4)	*T value*	*P* value
Glu	25.79 ± 4.63^***^	11.62 ± 3.88	*5.142*	*0.0006*
NE	6.88 ± 4.70^*^	0.10 ± 0.01	*2.816*	*0.0202*
DOPA	0.02 ± 0.007^**^	0.00 ± 0.00	*4.250*	*0.0021*
Tyr	7.04 ± 2.00^****^	30.8 ± 9.07	*6.905*	*<0.0001*
Thr	60.62 ± 26.17^*^	19.40 ± 6.88	*3.026*	*0.0143*
Ser	75.49 ± 21.52^**^	27.07 ± 1.44	*4.391*	*0.0017*
His	9.93 ± 1.63^*^	7.47 ± 0.53	*2.871*	*0.0184*
Ach	0.05 ± 0.02^**^	0.00 ± 0.00	*3.973*	*0.0032*
5-HT	1.26 ± 0.35^**^	0.37 ± 0.19	*4.708*	*0.0011*
HisA	0.55 ± 0.37^*^	0.06 ± 0.05	*2.582*	*0.0296*
Trp	11.57 ± 2.26^***^	17.85 ± 1.48	*4.925*	*0.0008*
Arg	12.27 ± 2.36^**^	17.62 ± 0.30	*4.402*	*0.0017*
Kyn	0.10 ± 0.06^***^	0.59 ± 0.22	*5.785*	*0.0003*
Lys	9.04 ± 8.25^****^	52.1 ± 3.97	*9.636*	*<0.0001*
Orn	4.41 ± 1.72^***^	10.88 ± 1.11	*6.586*	*0.0002*
Phe	8.37 ± 2.64^**^	16.57 ± 3.63	*4.348*	*0.0019*
Met	5.414 ± 0.99^**^	24.80 ± 14.80	*3.605*	*0.0057*
Asn	11.80 ± 3.66^**^	19.16 ± 1.16	*3.838*	*0.0040*

Compared with the ND group, *P<0.05, **P<0.01, ***P<0.001, ****P<0.0001. Glu, Glutamic acid; NE, Norepinephrine; DOPA, Dihydroxyphenylalanine; Tyr, Tyrosine; Thr, Threonine; Ser, Serine; His, Histidine; Ach, Acetylcholine; 5-HT, 5-Hydroxytryptamine; Trp, Tryptophan; Arg, Arginine; Kyn, Kynurenine; Lys, Lysine; Orn, Ornithine; Phe Phenylalanine; Met Methionine; Asn, Asparagine.

In the KD group, significant decreases were observed in the levels of tryptophan (Trp, *P* < 0.001), and kynurenine (Kyn, *P* < 0.001), lysine (Lys, *P* < 0.0001), tyrosine (Tyr, *P* < 0.0001), ornithine (Orn, *P* < 0.001), phenylalanine (Phe, *P* < 0.01), arginine (Arg, *P* < 0.01), methionine (Met, *P* < 0.01), and asparagine (Asn, *P* < 0.01) (**Table 2**).

## 4 Discussion

### 4.1 Effects of KD on cognition

A fundamental challenge in interpreting MWM data is distinguishing cognitive impairment from motor dysfunction. Comparable swimming speeds during visible platform training rule out the latter confound. It appears that the KD group performed in a contradictory manner, with long escape latency during acquisition coexisting with shorter target entry times and longer time spent in the target quadrant during probe trials. Longer escape latencies during acquisition suggest impaired striatal-dependent habit learning, whereas superior probe trial performance indicates enhanced hippocampal-dependent spatial memory. This pattern may suggest that equivalent behavioral outcomes can be supported by different neural substrates: when striatal function is compromised, hippocampal systems may dominate, yielding task-dependent advantages that appear contradictory under a unitary memory framework (Poldrack and Packard, 2003; Foerde et al., 2006). We speculate that KD may alter the balance between memory systems, potentially through changes in brain energy metabolism and neural plasticity. Our study aligns with preclinical evidence in epilepsy models, where the KD preserves spatial memory retrieval (Jiang et al., 2016; Wang et al., 2021) yet impairs MWM acquisition performance (Zhao et al., 2004). Marked study discrepancies stem not only from divergent epilepsy models but also from key KD intervention variables, including ketogenic ratio, animal developmental stage at initiation, and intervention duration. Wang et al (Wang et al., 2021). and Jiang et al (Jiang et al., 2016). used a clinical 4:1 KD in pentylenetetrazol (PTZ)-kindled rats with pre-kindling initiation, observing enhanced spatial memory acquisition and probe trial performance. In contrast, Zhao et al (Zhao et al., 2004). administered a high-ratio 8.6:1 low-protein KD to weanling rats immediately after lithium-pilocarpine induced SE for a 1-month prolonged intervention, documenting profound visual-spatial cognitive deficits and impaired brain growth.

Our 4-week KD protocol represents short-term metabolic adaptation. While brief KD exposure has been studied for seizure control, its acute cognitive effects in epilepsy models remain understudied, as prior research has predominantly examined prolonged interventions. This contrasts with our previous work (Jiang et al., 2016), where 8-week KD attenuated spatial and item memory impairment in PTZ-kindled rats, and with Wang et al (Wang et al., 2021), who reported that 12-week KD improved cognitive flexibility and spatial memory in epileptic models. Together, these findings suggest that short-term KD selectively enhances memory retention, whereas longer durations may be required for comprehensive cognitive protection.

### 4.2 Effects of KD on intestinal flora

After 4 weeks, the KD group’s gut microbiota showed a simpler composition, indicating less richness and diversity. It is consistent with the majority of studies, whether they examined short-term or long-term effects (Olson et al., 2018; Zhang et al., 2018).

The KD group exhibited a lower relative abundance of *Firmicutes*, a finding that aligns with previous research (Zhang et al., 2018; Wang et al., 2023). However, within the KD group, specific *Firmicutes genera*, including *Lactobacillus*, *Bifidobacterium*, and *Blautia*, were more abundant. Notably, *Lactobacillus* and *Blautia* enhance the intestinal barrier and reduce inflammation, helping to maintain gut health. We also found a significant reduction in *Spirochaetes* in the KD group. Reduced *Spirochaetes* have not been reported to affect cognition in epilepsy patients, but have been associated with cognitive decline in Alzheimer’s disease patients (Weber et al., 2023).

The decrease in *Bacteroidetes* is closely associated with cognitive impairment (Liang et al., 2022). In line with this, *Bacteroidetes* levels decreased in the present study, contrary to long-term KD findings (Wan et al., 2019). This may be consistent with our observed decrease in SCFAs, as *Bacteroidetes* are generally considered to exert anti-inflammatory effects by producing SCFAs. Additionally, *Parabacteroides* levels increased significantly, consistent with observations from other studies on very low-carbohydrate ketogenic diets (VLCKD) (Paoli et al., 2019). *Parabacteroides* has been identified as a producer of gamma-aminobutyric acid (GABA), a major inhibitory neurotransmitter that plays a crucial role in regulating brain states and cognitive functions (Liu et al., 2025).

Several “marker species” were found in KD gut microbiota, closely linked to KD’s antiepileptic and mood-improving effects (Nagao-Kitamoto et al., 2020; Mu et al., 2022; Zhou et al., 2023). However, there is a notable inconsistency between these marker species and the dominant genera typically found in the gut microbiota. Generally, marker species are associated with notable shifts under specific circumstances, whereas dominant species are associated with a healthy microbial community. Thereby, dominant genera like *Lactobacillus* and *Blautia* support general gut health by improving barrier function and reducing inflammation, whereas the marker species may specifically drive KD’s unique health benefits, such as seizure control and mood enhancement.

### 4.3 Effect of KD on SCFA

SCFAs are generated by gut microbes through the fermentation of dietary fiber. After short-term KD treatment, the levels of SCFAs such as acetate, propionate, and butyrate in rats’ feces decreased significantly, consistent with observations from short-term KD patients (Ferraris et al., 2021). We propose that two factors contribute to the significant drop in SCFA levels under a KD. First, the KD cuts dietary fiber intake, limiting substrates for gut microbiota to make SCFAs. The second cause is a change in the gut microbiota, which has fewer *Firmicutes* and *Bacteroidetes*. However, in contrast to our findings, Gong et al. (2021) reported increased SCFA concentration after 6 months of KD in children with drug-refractory epilepsy (Gong et al., 2021). According to this discrepancy, SCFAs underwent a biphasic change over time with KD, which may be caused by dietary substrates and microbes.

Patients with good outcomes had higher butyrate levels after KD, highlighting butyrate’s therapeutic role (Gong et al., 2021). Furthermore, SCFAs, particularly butyrate, improve cognitive function by increasing levels of neurotrophic factors and exhibit neuroprotective effects in neurodegenerative models (Dalile et al., 2019; Lee et al., 2020). Although SCFA levels were reduced in this study, rats on a KD exhibited superior cognitive performance, suggesting that additional mechanisms may contribute to the observed cognitive changes. With prolonged KD exposure, we hypothesize that SCFA levels may rise, suggesting SCFAs may still play a role in long-term cognitive benefits.

### 4.4 Effect of KD on neurotransmitters and amino acids

This study found that even short-term use of KD significantly altered neurotransmitter and amino acid levels in epileptic rats. KD’s anti-epileptic effects and cognitive function may be mediated by these changes (Kandimalla and Reddy, 2017).

This study showed a significant increase in Glu levels in the KD group. Glu mediates excitatory transmission and regulates synaptic plasticity in essential cognitive processes such as learning, memory, and attention (Albrecht and Zielinska, 2017). KD may alter the balance between memory systems, potentially through changes in brain energy metabolism and neural plasticity (Sedlak et al., 2019). In this study, after KD intervention, plasma levels of Arg decreased while Glu increased. As a result, KD may be associated with altered metabolism of Arg and Glu, enhancing their production and utilization (Albrecht et al., 1990).

Compared to the ND group, we observed a significant increase in NE and DOPA levels in the KD group. DOPA and NE are key neurotransmitters in the CNS that regulate cognitive functions. Specifically, NE enhances working memory and attention in the prefrontal cortex (PFC) via α-2A receptors (Ramos and Arnsten, 2007; Borodovitsyna et al., 2017), while DOPA modulates cognitive stability and flexibility through the balance of D1/D2 receptors (Riedel et al., 2022; Zhang Y. et al., 2025). It is interesting to note that KD effectively reduced the levels of Phe and Tyr precursors for NE and DOPA synthesis—consistent with previous findings in pediatric populations (Dahlin et al., 2005). It is possible that neurons may adapt enzyme activity and substrate affinity to convert limited Phe and Tyr into functional neurotransmitters under conditions of reduced substrate availability. Collectively, these observations raise the possibility that the protective effects of KD might be partially attributed to a mitigation of Phe elevation, which is known to be associated with white matter integrity and cognitive function (Thau-Zuchman et al., 2022).

In the present investigation, KD intervention increased 5-HT levels while suppressing Kyn levels. Trp metabolism involves either the Kyn pathway or the 5-HT pathway. Our results indicate a metabolic shift from the Kyn pathway toward the 5-HT synthesis pathway (Pérez-De La Cruz et al., 2007). It is important to make this shift since 5-HT regulates cognitive functions, including learning and memory, through the 5-HT6 receptor in the hippocampus (Dayer et al., 2015).

Here, we found that the KD significantly increased levels of Thr, His, and Ser. As Thr levels increase, acetylcholine (ACh) synthesis increases, improving learning and memory through M1 and 7-nicotinic acetylcholine receptors (7-nAChR) (Sangadi et al., 2024). The KD also raised His levels, which may protect cognition by regulating neuro-immunity through H1-H4 receptors and improving memory in epileptic rats (Parsons and Ganellin, 2006). Additionally, Ser may alleviate epilepsy-induced neuronal damage by promoting glial cell metabolism, thereby supporting cognitive function (de Koning et al., 2003). Other amino acids, such as Met and Orn, are also associated with cognition and metabolism, although their roles are less direct.

## 5 Conclusions

This study demonstrates that KD enhances cognitive function, which may be associated with changes in gut microbiota, SCFA metabolism, and neurotransmitter balance along the microbiota-gut-brain axis. However, the observed benefits may not solely reflect direct KD effects. Although behavioral testing was conducted during the latent phase post SE, seizure-related confounds were reduced but not eliminated. Additionally, the relatively small sample size and uneven group distribution, particularly in the context of microbiome analysis, should be considered when interpreting the findings. Moreover, central amino acids and neurotransmitters were not measured in this study, and long-term axis dynamics were not assessed. Future studies should monitor these parameters longitudinally in the same animals, while expanding sample size and optimizing group distribution to enhance statistical robustness.

## Data Availability

The datasets presented in this study can be found in online repositories. The names of the repository/repositories and accession number(s) can be found in the article/**Supplementary Material**.

## Supplementary material

Supplementary Figure 1 SUPPLEMENTARY FIGURE S1. (A) Levels of blood glucose, and (B) Levels of β-hydroxybutyrate (BHB) in either ketogenic diet (KD) or normal diet (ND) (n = 7 per group). ***p < 0.001 compared with ND.

SUPPLEMENTARY FIGURE S2 Seizure burden during status epilepticus in a lithium-pilocarpine model. (A) Latency to seizure, (B) total cumulative duration of seizures, and (C) total cumulative duration of generalized seizures (GS). Data are presented for both ketogenic diet (KD) and normal diet (ND) groups (n = 7 per group).

SUPPLEMENTARY FIGURE S3 **Morris water maze (MWM) test**. (A) Swimming speed during the visible platform trial on Day 0. (B) Latency to reach the target platform and latency of first entry into the target quadrant during hidden-platform acquisition training (Days 1–4). (C) Spatial probe trial on Day 5 showing swimming speed, number of target crosses, and percentage of time spent in each quadrant of the MWM (n = 7 per group). Abbreviation: Q, quadrant.*P < 0.05 indicate significant differences between ketogenic diet (KD) and normal diet (ND) groups. #P < 0.05 and ##P < 0.01 indicate significant differences compared with Day 1 within the same group.
