# Supplementary figures and images for: From gut to brain: short-term ketogenic diet alleviates status epilepticus-induced cognitive deficits in rats
Source: Front Physiol. 2026 Apr 28;17:1752371. doi: 10.3389/fphys.2026.1752371 (PMC13160795; doi:10.3389/fphys.2026.1752371)

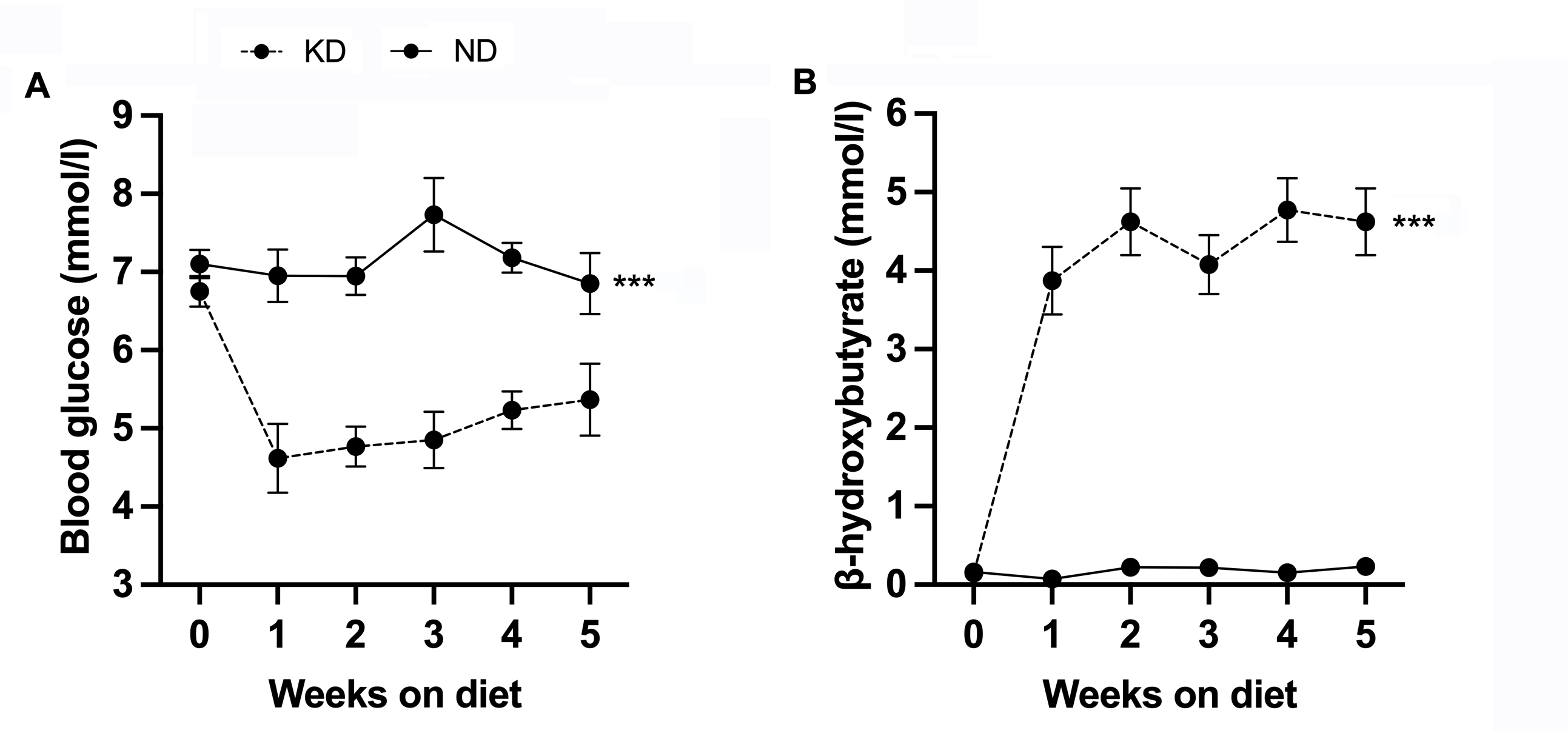

Supplement: Supplementary Figure 1 — SUPPLEMENTARY FIGURE S1. (A) Levels of blood glucose, and (B) Levels of β-hydroxybutyrate (BHB) in either ketogenic diet (KD) or normal diet (ND) (n = 7 per group). ***p < 0.001 compared with ND. [file Image1.tiff]

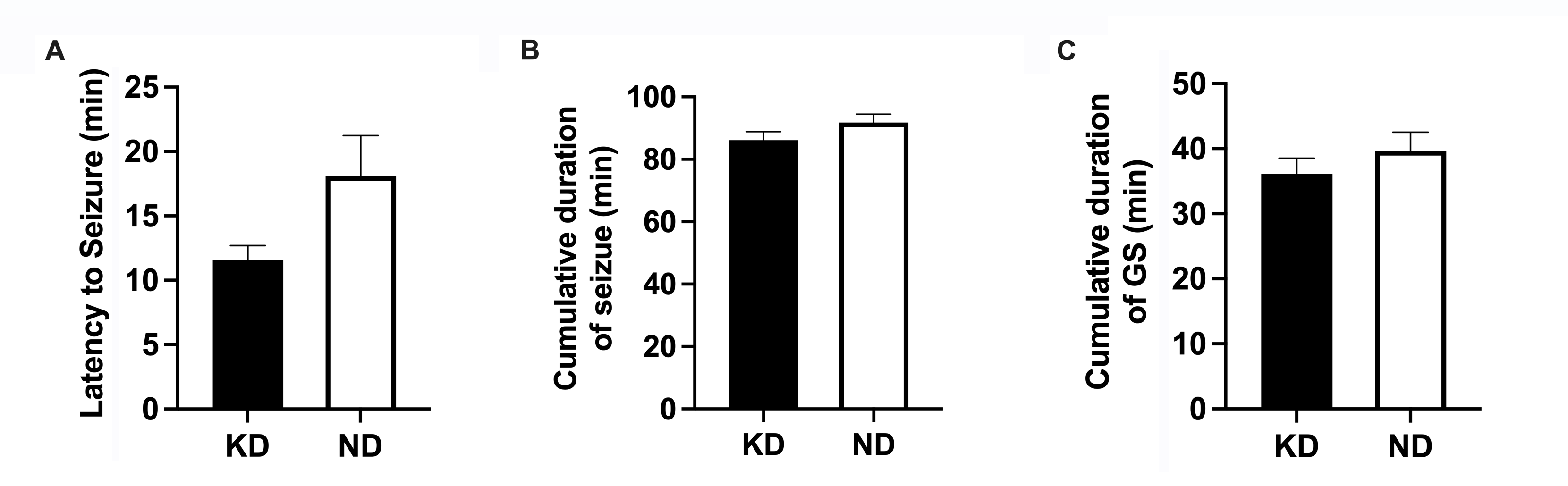

Supplement: SUPPLEMENTARY FIGURE S2 — Seizure burden during status epilepticus in a lithium-pilocarpine model. (A) Latency to seizure, (B) total cumulative duration of seizures, and (C) total cumulative duration of generalized seizures (GS). Data are presented for both ketogenic diet (KD) and normal diet (ND) groups (n = 7 per group). [file Image2.tiff]

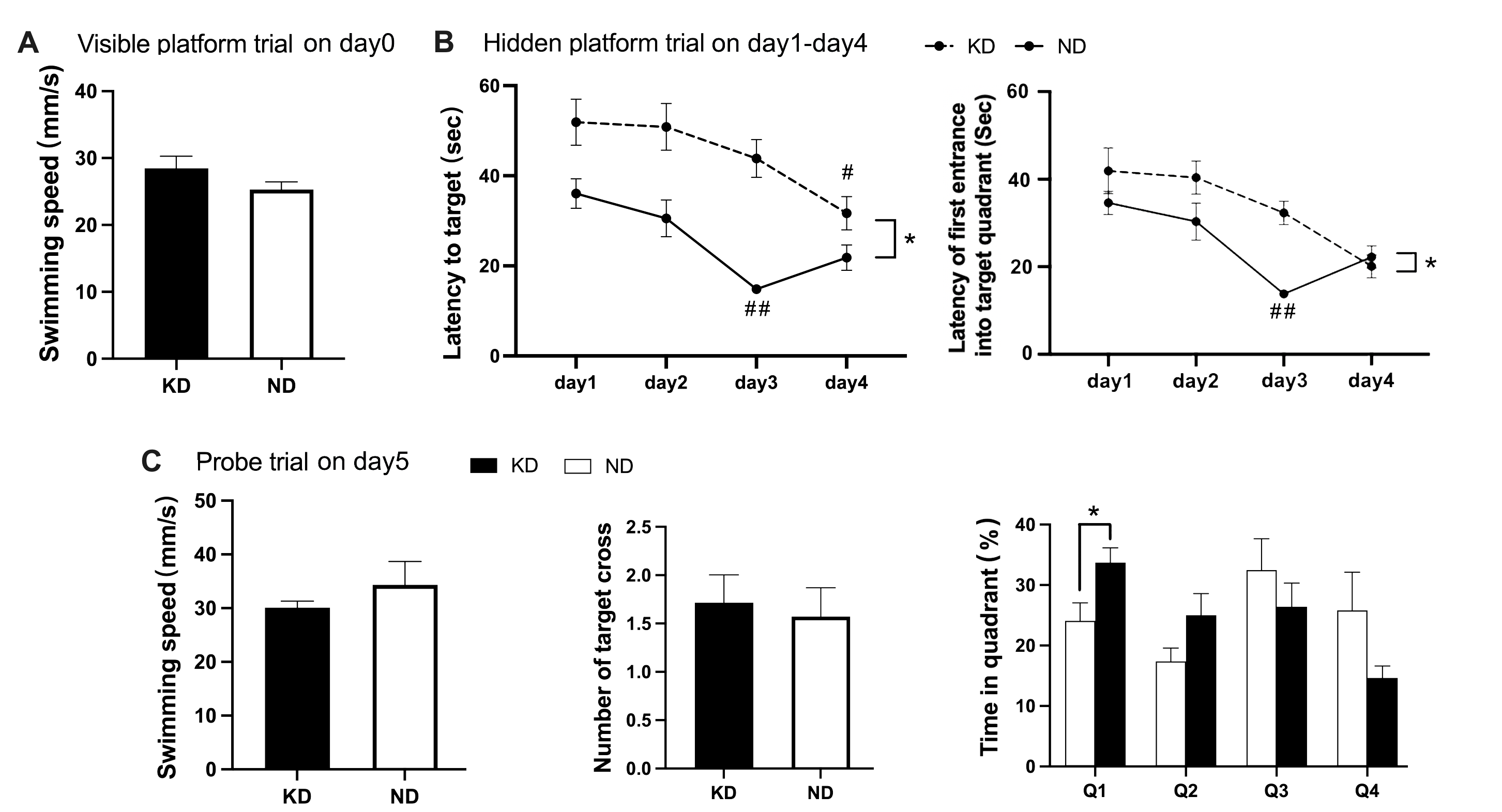

Supplement: SUPPLEMENTARY FIGURE S3 — Morris water maze (MWM) test. (A) Swimming speed during the visible platform trial on Day 0. (B) Latency to reach the target platform and latency of first entry into the target quadrant during hidden-platform acquisition training (Days 1–4). (C) Spatial probe trial on Day 5 showing swimming speed, number of target crosses, and percentage of time spent in each quadrant of the MWM (n = 7 per group). Abbreviation: Q, quadrant.*P < 0.05 indicate significant differences between ketogenic diet (KD) and normal diet (ND) groups. #P < 0.05 and ##P < 0.01 indicate significant differences compared with Day 1 within the same group. [file Image3.tiff]
