# Supplementary material for: From gut to brain: short-term ketogenic diet alleviates status epilepticus-induced cognitive deficits in rats
Source: Front Physiol. 2026 Apr 28;17:1752371. doi: 10.3389/fphys.2026.1752371 (PMC13160795; doi:10.3389/fphys.2026.1752371)
Supplement: Supplementary file 4 [file SupplementaryFile1.docx]

**Morris Water Maze (MWM) test**

The MWM apparatus was provided by the Animal Center of Zhejiang Chinese Medical University. All procedures were conducted in accordance with institutional guidelines. A circular pool (200 cm in diameter) filled with water (22 ± 1 °C) made opaque with powdered milk was used, with fixed reference objects placed around the perimeter. The pool was divided into four quadrants (NE, NW, SW, and SE), designated as quadrants 1, 2, 3, and 4, respectively, with a circular target platform (10 cm in diameter) located in quadrant 1 (NE).

The MWM test consisted of a visible platform trial on day 0, followed by 4 days of acquisition training (hidden platform trials, days 1–4), and a spatial probe trial on day 5.

**Visible platform trial (day 0):** The platform was positioned 1 cm above the water surface. Rats were placed in the water facing the pool wall in randomly assigned quadrants. Rats that find the platform are allowed to remain there for 10 seconds; if the platform is not found within 60 seconds, the rat is gently guided to it, and the latency is recorded as 60 seconds. Each rat was measured for its swimming speed.

**Hidden platform trials (days 1–4):** The platform was hidden 1 cm below the water surface. The training was conducted over four consecutive days with four trials per day and one hour between trials. For all trials, escape latency was measured.

**Spatial probe trial (day 5):** The platform was removed, and rats were released from a random quadrant. The swimming speed, the latency of first entrance into the target square, the time spent in, and the number of crossings into the target quadrant were recorded over 60 s.

Data were recorded and analyzed by Smart 3.0 video tracking system (RWD Life Science, Shenzhen, China).
